# Silver multilayer coating on orthopedic implant material of different alloys and surfaces significantly reduces bacterial colonization

**DOI:** 10.3389/fcimb.2025.1707694

**Published:** 2025-11-26

**Authors:** Lydia T. D. Speijker, Janine Fechter, Rainer Bargon, Jozef Dingemans, Jacobus J. Arts, Paul H. M. Savelkoul, Inge H. M. van Loo

**Affiliations:** 1 Department of Medical Microbiology, Infectious Disease and Infection Prevention, Maastricht University Medical Centre, Maastricht, Netherlands; 2 Care and Public Health Research Institute (CAPRHI), Maastricht University, Maastricht, Netherlands; 3 Department of Research and Development, Aesculap AG, Tuttlingen, Germany; 4 Clinical Laboratory of Molecular Microbiology, Jessa Hospital, Hasselt, Belgium; 5 Laboratory for Experimental Orthopaedics, Department of Orthopaedic Surgery, Maastricht University Medical Centre, Maastricht, Netherlands; 6 Department of Biomedical Engineering, Orthopaedic Biomechanics, Eindhoven University of Technology, Eindhoven, Netherlands

**Keywords:** prosthetic joint infection, silver, antibacterial agents, *in vitro*, prevention of bacterial colonization, materials

## Abstract

**Introduction:**

Prosthetic joint infections (PJI) affect 1-3% of prosthetic joint replacements, frequently linked to biofilm formation on implant surfaces. PJIs account for 13.0-31.3% of all revision surgeries. As treatment is not always successful, prevention remains critical. Currently, silver based antimicrobial coatings are justified in select high-risk arthroplasty cases, restricted to metal surfaces. An antibacterial silver multilayer coating (SML) has been developed for implant materials: titanium alloy (Ti) and cobalt-chromium-molybdenum alloy (CoCr), often used in hip or knee prostheses. The SML coating is intended for revision implants, which are often required due to infection related implant failure. This study investigates the antibacterial performance of the SML coating across different surfaces and implant materials, using multiple bacterial strains not previously investigated to this extent.

**Methods:**

Antibacterial efficacy of the SML coating was assessed by quantifying colony forming units (CFU) reduction on Ti6Al4V and Co28Cr6M discs with three different surfaces (polished and two grades of corundum blasted (CB)). *In vitro* standardized testing followed ISO 22196, JIS Z-2801, and ASTM E-2180 standards using American Type Culture Collection (ATCC) strains *Pseudomonas aeruginosa* ATCC15442, *Staphylococcus aureus* ATCC6538p, *Staphylococcus epidermidis* ATCC35984, *Pseudomonas aeruginosa* ATCC15442, and *Escherichia coli* ATCC8739. Two groups were tested: non-SML-coated samples and SML-coated samples. After 24 hours incubation in viscous nutrient broth at 37°C, viable bacteria were quantified per disc after sonication in neutralizing broth and CFU enumeration.

**Results:**

Across all materials and strains, the SML coating achieved >99.2% and >0.9-4.0 CFU log_10_ reduction in viable bacteria compared to the non-SML-coated controls. Material-dependent effects were observed for each of the bacterial species analyzed. *S. aureus* and *E. coli* exhibited more CFUs on Ti than on CoCr. The CoCr CB surface yielded the lowest level of bacterial growth for *P. aeruginosa* ATCC15442, whereas *S. epidermidis* colonized the Ti CB surface more extensively.

**Discussion and conclusion:**

These findings demonstrate a thorough and broad-spectrum antibacterial activity of the SML coating across diverse implant materials and surface textures. Future studies will focus on testing clinical PJI isolates in both *in vitro* and *in vivo models* to further evaluate the translational potential of the SML coating for prevention of bacterial colonization in joint arthroplasty.

## 1 Introduction

Prosthetic joint infections (PJI) form a severe complication following joint arthroplasty, affecting approximately 1.4-2.8% of primary prosthetic joint replacement patients (Kim et al., 2020; Kheir et al., 2021; Bozzo et al., 2022; Weinstein et al., 2023). They account for 13.0-31.3% of all revision surgeries, making them one of the leading causes of implant failure (Bozic et al., 2010; Perfetti et al., 2017; Patel et al., 2023). Bacteria adhere to the implant surface, allowing them to form biofilms. Once established on the implant surface, biofilms protect bacteria from host immune defenses and antibiotic treatment, resulting in difficulty of eradicating the biofilm necessitating additional revision surgeries (Staats et al., 2021; Visperas et al., 2022). As therapeutic success remains limited, prevention of bacterial adhesion and biofilm formation represents a clinical strategy to reduce infection risk (Ricciardi et al., 2020).

A variety of materials are employed in orthopedic prosthesis, including titanium alloys (Ti), cobalt-chromium-molybdenum alloys (CoCr), stainless steel, and zirconia (Merola and Affatato, 2019). Surface modifications further influence implant performance. Corundum blasted (CB) surfaces, for example, have been reported to enhance bone formation, particularly on femoral stems, while polished surfaces are typically found on femoral heads (Robinson et al., 1996; Allen and Raeymaekers, 2021). While this rougher surface might be favorable for osseointegration, rough surfaces have been reported to facilitate biofilm growth better than polished surfaces, possibly due to the protection from shear forces (McGaffey et al., 2019; Kuik et al., 2025).

Antimicrobial coatings may preclude attachment and biofilm formation due to anti-adhesion, contact-killing or releasing-type strategies, thereby preventing infection (Cloutier et al., 2015). Silver is generally known to have antimicrobial properties. Therefore silver based antimicrobial coatings have attracted considerable interest as potential preventive strategies for bacterial colonization on orthopedic implants (Sim et al., 2018). Currently, their clinical use seemed to be restricted to high-risk arthroplasty cases, and no silver coatings are commercially available for cementless implantation of prosthetic joints which fully covers the femoral stem (Gruner et al., 2025). A silver containing hydroxyapatite-coated total hip prosthesis AG-PROTEX (KYOCERA Inc., Kyoto, Japan) is commercially available in Japan (Kawano et al., 2023). Different silver-coated megaprostheses are commercially available in the EU, such as silver-coated MUTARS^®^ (Implantcast, Buxtehude, Germany), METS^®^ + Agluna^®^ (Modular Endoprosthetic Tumour System, Stanmore Implants Worldwide, Elstree, UK and Accentus Medical Ltd, Oxfordshire, UK), MegaC^®^ + PorAg^®^ (Waldemar Link GmbH & Co. KG, Hamburg, Germany). However is it reported that the prosthesis is not fully coated with silver, the articulating surface and the prosthetic stems remain uncoated (Fiore et al., 2021). As a result, antimicrobial protection might be incomplete, leaving uncoated areas susceptible to bacterial colonization.

Case reports suggest that silver-coated implants can aid in managing PJI, with systemic silver concentrations remaining below detection limits in treated patients (Alt et al., 2019; Ismat et al., 2020; Alt, 2022; Alt et al., 2022; Lenz et al., 2023; Gruner et al., 2025). Building on these insights, an antibacterial silver multilayer coating (SML, HyProtect™, Bio-Gate, Nuernberg, Germany) has been developed for application on both metallic and polymeric implant materials (Fabritius et al., 2020). This ultra-thin coating consists of a ~90 nm layer where silver aggregates are embedded within a polysiloxane (SiO_x_C_y_) matrix (Khalilpour et al., 2010; Fabritius et al., 2020). The safety and antibacterial properties of this SML coating have been shown using *in vitro* and *in vivo* antimicrobial activity assays, ASTM E-2810-01, ASTM E-2801–7 and JIS Z 2801:2000, and cytotoxicity assays, ISO 10993-6 (Khalilpour et al., 2010; Azab et al., 2016; Fabritius et al., 2020; Stein et al., 2020). However, the roughness of the surface and porosity can affect the coating surface area which might influence the adherence ability of bacteria (Zheng et al., 2021).

An important consideration in the translation of antibacterial coatings to clinical use is biocompatibility. Previous *in vivo* analyses have shown no systemic silver release and only trace accumulation in local tissues, supporting the biosafety of the SML coating (Fabritius et al., 2020). This indicates that osteoblasts proliferation is not at risk, similarly to another study which showed that the silver coating used was biocompatible in regard to proliferation of fibroblasts and preosteoblasts (Choi et al., 2023). Grüner et al., 2025, demonstrated successful osseointegration of prosthetic devices with an ultrathin SML coating over a three year follow up period, further supporting its potential clinical applicability (Gruner et al., 2025).

To date, the antibacterial properties of the SML coating across different implant materials and surface properties have not been systemically investigated. Previously, only Ti CB discs have been assessed with three different bacterial strains also using internationally standardized test methods (van Hoogstraten et al., 2024). Using similar methods, this study therefore aims to evaluate the antibacterial performance of the SML coating on Ti and additionally CoCr materials with both polished and CB surfaces. An additional aim of this study is to assess the Ti and CoCr material dependent effects on bacterial growth.

## 2 Materials and methods

### 3.1 Bacterial strains and antibacterial activity test

Both sterile Ti (Ti6Al4V) and sterile CoCr discs (Co28Cr6Mo) (Ø14 mm x 5 mm, Aesculap AG, Tuttlingen, Germany) were used for this study. These discs contained different surfaces: polished for both Ti and CoCr (surface roughness average (Ra) 0.1 μm) and CB Ra 5-7 μm for Ti and Ra 4-7 μm for CoCr. Two experimental groups were included: non-SML-coated (“uncoated”), and discs coated with the SML coating (“SML-coated”), applied as previously described (van Hoogstraten et al., 2024).

Bacterial strains were selected based on clinical relevance, relevant literature, and in line with international standards (WHO, JIS 2801): *S. aureus* ATCC6538P (JIS Z 2801, 2010; ISO 22196, 2011), *S. epidermidis* ATCC 35984 (Polonio et al., 2001; Skiba-Kurek et al., 2021; Tambone et al., 2021), *E. coli* ATCC8739 (JIS Z 2801, 2010; ISO 22196, 2011), and *P. aeruginosa* ATCC15442 (ASTM E 2180-24, 2024). Each strain was tested independently. For each test condition (material and surface type), three SML-coated discs were tested, three uncoated discs were included for immediate retrieval and as a positive control and three uncoated discs were used as a positive control as well and retrieved after 24 hours. All experiments were conducted three times and in triplicate.

Since *P. aeruginosa* ATCC15442 is an environmental strain, four clinical *Pseudomonas aeruginosa* (*P. aeruginosa*) strains isolated from prosthetic joint infections (PJIs, “PJI1-PJI4”), along with one fracture related infection (FRI, “FRI1”) and two reference strains (*P. aeruginosa* ATCC15442 and ATCC15692) were included to evaluate their biofilm properties. From these 4 clinical isolates, the biofilm properties were evaluated and the clinical strain that formed the most biomass after 24H incubation was used for further evaluation.

This clinical isolate was evaluated on Ti polished, CoCr polished, and CoCr CB. For each of these surfaces, six uncoated discs were used: three retrieved immediately after inoculation, and three after 24 hours of incubation. In addition, three SML-coated discs were included for Ti polished and CoCr polished, while to SML-coated discs were available for the CoCr CB surface.

Antibacterial efficacy of the SML coating was assessed according to the principles of the International Organization for Standardization (ISO) 22196:2007 (ISO 22196, 2011), Japanese Industrial Standard (JIS) Z 2801:2000 (JIS Z 2801, 2010), and American Society for Testing and Materials (ASTM) E-2180-24 (ASTM E 2180-24, 2024) test standards.

### 3.2 Attachment assays

For additional confirmation of the antibacterial efficacy of the SML coating and to assess the potential surface-dependent effects, one clinical *P. aeruginosa* isolate from a PJI was included. Attachment assays were performed to determine the clinical strain that formed the most biomass after 24 hours incubation, similarly performed as previously described (Dingemans et al., 2019). All seven *P. aeruginosa* isolates/strains were cultured overnight at 37°C in 96-well microtiter plates under shaking conditions (180 rpm) to a target concentration of approximately 10^7^ CFU/ml. Subsequently, 50 µl of a 0.1% weight/volume crystal violet solution was added to each well, followed by incubation for 15 minutes at 37°C with continuous shaking (180 rpm). After incubation, wells were washed three times with distilled water to remove non-adherent cells and excess dye. The retained crystal violet was solubilized in 96% ethanol, and absorbance was measured at 570 nm (OD_570_) to quantify bacterial attachment.

### 3.3 Bacterial growth and viable cell count

Discs were placed in a 12-wells plate (Fisher Scientific), pre-wetted with PBS, and inoculated with a bacterial suspension (target concentration of 6*10^5^ colony forming units (CFU)/ml) in 0.2% nutrient broth (Millipore^®^). To increase viscosity and to ensure uniform contact between the inoculum and test surface, 3g/L lukewarm agar (Thermo Scientific) was added to the medium. Immediately after inoculation, half of the uncoated discs were added in a falcon tube (Greiner Bio-One), respectively, containing 5 mL soybean casein digest broth with lecithin and polyoxyethylene sorbitan monooleate and tween-20 (SCDLP broth; Millipore^®^). Discs were then sonicated in an ultrasonic cleaning bath (Branson) for 10 minutes to dislodge adherent bacteria.

Following sonication, the resulting suspensions were serially diluted and 100 μL of this sonication fluid was plated on Columbia III agar plates containing 5% sheep blood (BD). Plates were incubated for 24 hours at 37°C before CFU enumeration. The remaining SML-coated and uncoated samples were incubated under humidified conditions at 37°C for 24 hours. After incubation, these samples were similarly transferred to 5 mL SCDLP broth and sonicated to remove surface associated bacteria. The sonicates were then diluted and plated under the same conditions for CFU quantification.

As all discs had identical surface areas (cm^2^), viable bacterial counts are reported as “CFU on sample”. The CFU on sample was calculated by multiplying the counted CFUs by a hundred, as well as the dilution factor to get to the countable CFUs, and the volume in mL of SCDLP broth added to the specimen, and all this was divided by the surface area. CFU counts from samples immediately retrieved after inoculation were used to define the starting inoculum. The limit of detection was set at 50 CFUs, due to one colony being the lowest countable number of colonies in 100 μL, resulting in 50 CFUs per sample and per 5 mL. Data are presented as means ± standard error of the mean (SEM) from triplicate samples across three independent experiments.

The antibacterial effect was assessed by calculating the log_10_ geometric mean per group of CFU counts on the sample which was incubated for 24 hours, by adding the three different log_10_ counts and dividing it by 3. Results were calculated as percentage reduction using the antilogarithm of the geometric mean of non-SLM CFUs on the sample which was incubated for 24 hours as reference. Similarly to CFU log_10_ reduction, antibacterial activity (R value) was calculated by subtracting the average log_10_ cells/cm^2^ of the treated specimens after 24 hours of incubation by average log_10_ cells/cm^2^ of the untreated specimens.

### 3.4 Visualization of discs with scanning electron microscopy

To visualize bacterial growth on the disc surfaces, *E. coli* ATCC8739 was cultured (target concentration of 6*10^5^ colony forming units (CFU)/ml) for 24 hours on both SML-coated and uncoated discs. Due to limited SML-coated disc availability, only SML-coated discs inoculated with *E. coli* ATCC8739 were imaged (n=3 per surface). Uncoated discs of all types and surfaces were imaged for all previously mentioned bacteria (*S. aureus* ATCC6538P, *S. epidermidis* ATCC 35984, *P. aeruginosa* ATCC15442, and *E. coli* ATCC8739) (n=1 due to uncoated discs availability). After inoculation, 24 hours of incubation at 37 °C, and washing in PBS, discs were subsequently fixed in 2.5% glutaraldehyde (Sigma-Aldrich). Following fixation, the discs were washed in 0.1M phosphate buffer and post-fixed in 1% osmium tetroxide (Van Loenen Instruments). Discs were rinsed again with 0.1 M phosphate buffer, dehydrated in a graded ethanol series (70%, 90%, 100%, 100%, 30 minutes per step), and dried twice using hexamethyldisilazane (Sigma-Aldrich). After drying, the discs were mounted on aluminum stubs (Van Loenen Instruments) using carbon adhesive stickers and subsequently carbon sputter coated with a 5 nm layer of carbon (Leica, Amsterdam, The Netherlands). Two scanning electron microscopy (SEM) images were obtained per disc, capturing representative areas of the surface using a Jeol JSM-IT200 (InTouchScope™, Freising, Germany).

### 3.5 Statistical analysis

Data analysis was performed using Microsoft Excel 2016, GraphPad Prism software version 5, and IBM SPSS statistics version 28. CFU count data are inherently non-normally distributed, as they frequently include zero values and tend to be highly skewed (Olsen, 2003; Gonzales-Barron et al., 2010). Therefore, non-parametric tests were performed. Attachment assay results were analyzed using Mann-Whitney U tests. To evaluate the effect of the SML coating on viable bacterial counts, Mann-Whitney U tests were applied. The influence of surface type and roughness on viable counts was assessed using Kruskal-Wallis tests with *post hoc* analysis (Mann-Whitney U test). A *p* value of <0.05 was considered statistically significant for all comparisons.

## 4 Results

### 4.1 Attachment properties of clinical PJI *P. aeruginosa* strains

**Figure 1:**
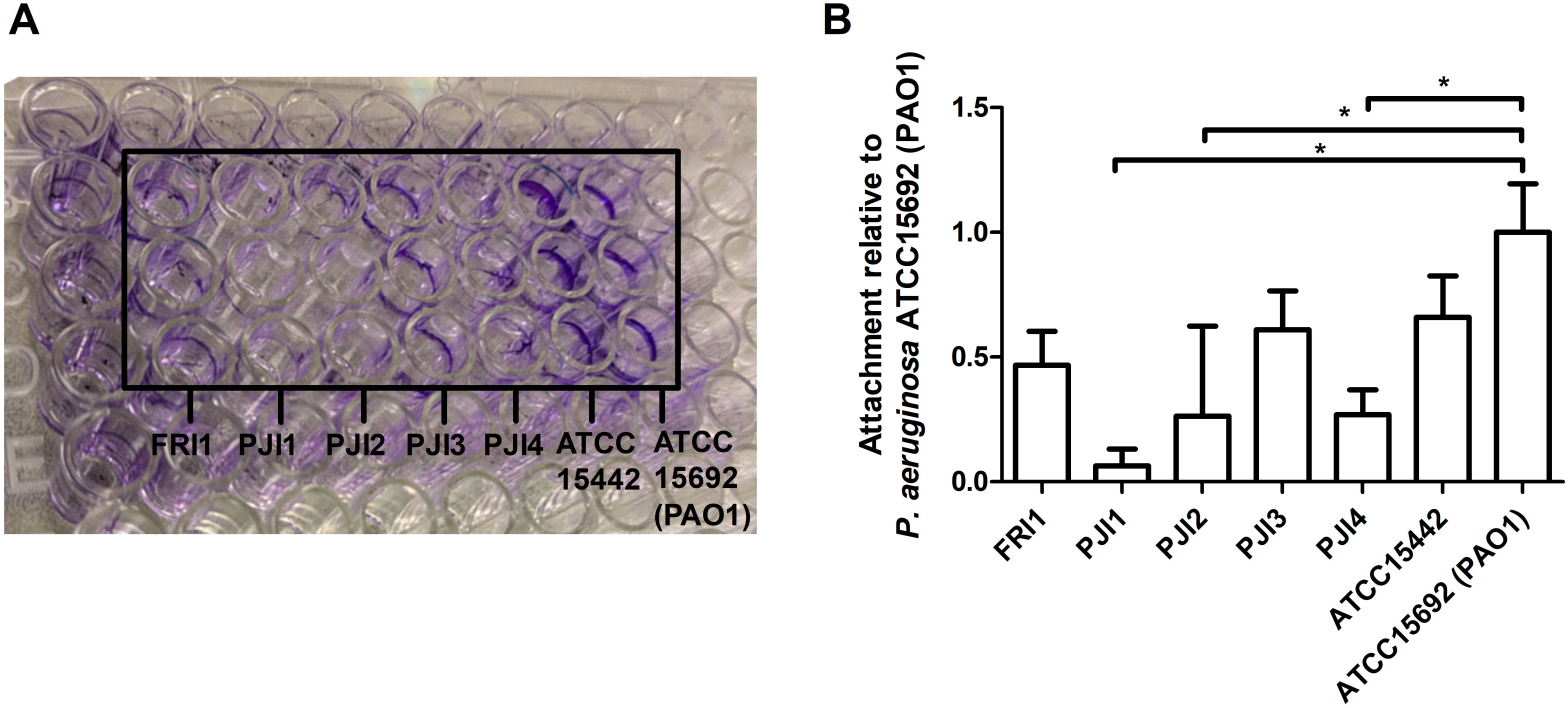
Attachment assay of *P. aeruginosa* strains, including different clinical strains from isolated from a FRI (n=1), PJIs (n=4), and ATCC strains ATCC15442 and ATCC15692. Visual inspection of the biofilm rings are seen **(A)**. Quantification of biofilm formation based on OD_570_ measurements **(B)**. Statistical comparisons were made using a student’s t-test relative to the ATCC15692 (PAO1) strain. FRI, fracture related infection; PJI, prosthetic joint infection; ATCC, American Type Culture Collection. *p<0.05.

Attachment assays were performed to identify the most potent biofilm-forming *P. aeruginosa* strain isolated from PJI-related infections. This assay determines the amount of biomass produced, including dead and live cells. Visually, the PJI3 and FRI1 clinical isolate, along with reference strains ATCC15442 and ATCC15692 (PAO1), exhibited more intense and thicker colored biofilm rings compared to the other clinical isolates (**Figure 1A**). Quantitative assessment of crystal violet staining confirmed that PJI3, FRI1, and ATCC15442 showed comparable attachment levels as ATCC15692 (PAO1), a known strong biofilm former (Berkl et al., 2022), with no statistically significant differences between these strains (**Figure 1B**). In contrast, the remaining clinical isolates demonstrated significantly lower attachment levels as ATCC15692. Based on these results, both ATCC15442 and PJI3 were selected to determine the antibacterial efficacy of the SML coating and to assess potential surface-dependent effects.

### 4.2 Effects of SML coating on bacterial survival

**Figure 2:**
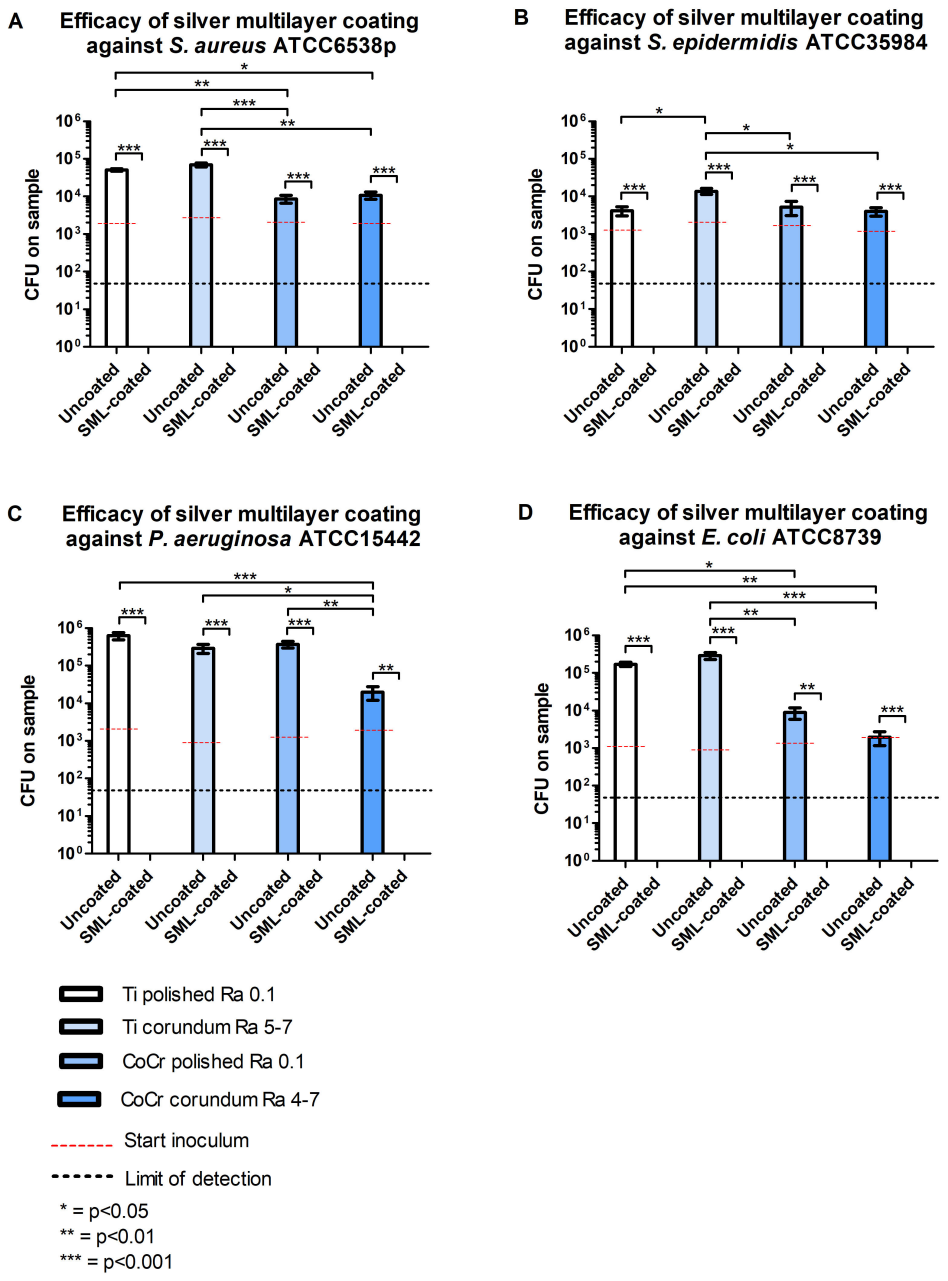
Efficacy of SML coating, surface, and material on the reduction of bacterial growth of four bacterial species: *S. aureus* ATCC6538p **(A)**, *S. epidermidis* ATCC35984 **(B)**, *P. aeruginosa* ATCC15442 **(C)**, *E. coli* ATCC8739 **(D)**. Bars represent the mean colony forming units (CFU) of three independent experiments performed in triplicate (n=9 per condition). Error bars indicate the standard error of the mean. Statistical analysis was performed using the Kruskal-Wallis test, with *post hoc* analysis (Mann-Whitney U test). *p<0.05, **p<0.01, ***p<0.001. SML, silver multilayer; Ti, titanium alloy; CoCr, cobalt-chromium-molybdenum alloys; Ra, surface roughness average; ATCC, American Type Culture Collection.

All SML-coated discs (Ti and CoCr) showed <50 CFUs per sample for all strains, indicating a reduction in bacterial load of more than >99.2% compared to uncoated controls (*p* < 0.01 for all species; **Figure 2**; **Supplementary Figure S1**). More specifically, *S. aureus* showed an antibacterial activity (R) and CFU log_10_ reduction greater than 2.6 ± 0.3 (99.9%) across all surfaces. For *S. epidermidis* ATCC35984, *E. coli* ATCC8739, *P. aeruginosa* ATCC15442, and the clinical *P. aeruginosa* PJI3 strain, antibacterial activity (R) and reductions of respectively more than 1.9 ± 0.2 (99.7)%, 2.6 ± 0.6 (99.5)%, 3.2 ± 0.4 (99.3%), and 2.1 ± 0.8 (99.2%) were observed (**Table 1**; **Supplementary Table S1**).

**Table 1:** Overview of the bacterial reduction (%) and antibacterial activity (R) for each tested microorganisms and disc type.

Micro-organism	Disc type	Reduction in viable bacteria (uncoated vs. SML-coated) in %	R (antibacterial activity)
*S. aureus* ATCC6538P	Ti polished Ra 0.1 μm	100.0	>3.0
	Ti CB Ra 5-7 μm	100.0	>3.1
	CoCr polished Ra 0.1 μm	>99.9	>2.0
	CoCr CB Ra 4-7 μm	>99.9	>2.1
*S. epidermidis* ATCC35984	Ti polished Ra 0.1 μm	>99.9	>1.8
	Ti CB Ra 5-7 μm	100.0	>2.4
	CoCr polished Ra 0.1 μm	>99.7	>1.7
	CoCr CB Ra 4-7 μm	>99.8	>1.7
*P. aeruginosa* ATCC15442	Ti polished Ra 0.1 μm	100.0	>4.0
	Ti CB Ra 5-7 μm	100.0	>3.6
	CoCr polished Ra 0.1 μm	100.0	>3.8
	CoCr CB Ra 4-7 μm	>99.3	>1.8
*E. coli* ATCC8739	Ti polished Ra 0.1 μm	100.0	>3.5
	Ti CB Ra 5-7 μm	100.0	>3.7
	CoCr polished Ra 0.1 μm	>99.5	>1.8
	CoCr CB Ra 4-7 μm	>99.5	>1.3

Values represent the mean reduction in viable bacteria of three independent experiments performed in triplicate (n=9 per condition), comparing uncoated and SML-coated discs. SML, silver multilayer; Ti, titanium alloy; CoCr, cobalt-chromium-molybdenum alloys; CB, corundum blasted; Ra, surface roughness average; ATCC, American Type Culture Collection.

### 4.3 Effect of surface type on bacterial growth

For *S. aureus*, significantly higher CFU counts were observed on Ti (Ti polished: 5.1*10^4^ CFU, Ti CB: 6.9*10^4^ CFU) compared to CoCr (CoCr polished: 8.7*10^3^ CFU, CoCr CB: 1.1*10^4^ CFU, *p* < 0.05, **Figure 2A**). Similarly, *E. coli* showed greater growth on Ti (polished: 1.7*10^5^ CFU, CB: 2.9*10^5^ CFU) compared to CoCr material (polished: 8.8*10^3^ CFU, CB: 1.9*10^3^ CFU, *p* < 0.05, **Figure 2D**). In the case of *S. epidermidis*, significantly more growth was only observed on Ti CB Ra 5-7 μm (1.4*10^4^ CFU) compared to the other tested surfaces (Ti polished: 4.2*10^3^ CFU, CoCr polished: 5.2*10^3^ CFU, CoCr CB: 4.0*10^3^CFU, *p* < 0.05, **Figure 2B**). For *P. aeruginosa*, the lowest bacterial counts were observed on the CoCr CB surface (2.0*10^4^ CFU) compared to all the other tested surfaces (Ti polished: 6.3*10^5^ CFU, Ti CB 5-7: 2.9*10^5^ CFU, CoCr polished: 3.7*10^5^ CFU, *p* < 0.05, **Figure 2C**).

### 4.4 Scanning electron microscopy

**Figure 3:**
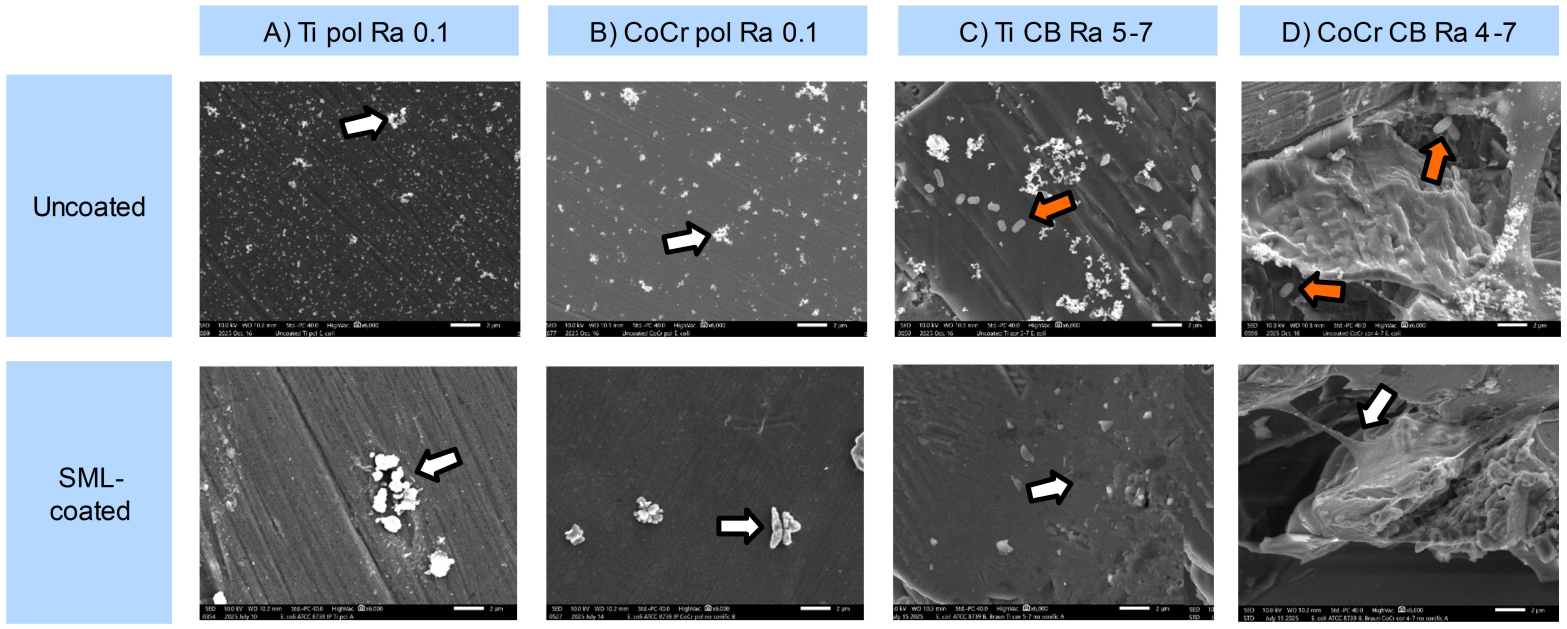
Scanning electron microscopy (SEM) visualization of *E. coli* ATCC8739 survival on different materials **(A–D)** and both uncoated (n=1) and SML-coated (n=3 per surface) samples to assess the efficacy of the SML coating. Orange arrows indicate bacterial cells, while white arrows indicate debris or remnants of bacterial cells or biofilm matrix. In **(D)**, seemingly extracellular matrix is visible, suggesting residual biofilm components. Images were acquired at 6000X magnification, with scale bars representing 2 µm. SML, silver multilayer; Ti, titanium alloy; CoCr, cobalt-chromium-molybdenum alloys; Ra, surface roughness average; CB, corundum blasted; pol, polished; Ra, surface roughness average.

Both uncoated and SML-coated discs with CB and polished surfaces were visualized by SEM following inoculation and incubation of *E. coli* (**Figure 3**). Uncoated CB control samples demonstrated clear *E. coli* colonization (**Figures 3C, D**, row uncoated), indicated by an orange arrow in the figures. Uncoated polished samples showed no visible colonization for the other tested bacteria (*S. aureus* ATCC6538P, *S. epidermidis* ATCC 35984 and *P. aeruginosa* ATCC15442, **Supplementary Figures S2**, **S3**). No bacterial colonization was observed in the SML-coated samples on any of the surfaces (**Figures 3A–E**, row SML-coated), nor on the uncoated polished surfaces (**Figures 3A, B**, row uncoated). Occasional debris or remnants of bacterial cells were present on polished uncoated samples, as well as all SML-coated samples, indicated by the white arrows in the figures.

## 5 Discussion

In the present study, antibacterial efficacy of SML coating on Ti and CoCr materials with different surface roughness was evaluated. The silver coating exhibited significant antibacterial activity against both Gram-positive and Gram-negative species, including *S. aureus*, *S epidermidis, P. aeruginosa* (environmental and clinical strains) and *E. coli*, with a >99.2% and >0.9-4.0 CFU log_10_ reduction in bacterial counts. Therefore, antimicrobial surface modifications of implant materials represent a promising strategy to reduce infection risk of prosthetic arthroplasty.

A similar study using a lower roughness of Ti CB by Van Hoogstraten et al., 2024 (van Hoogstraten et al., 2024) reported residual *E. coli* CFUs exceeding 10^7^ CFUs on Ti CB control discs for *E. coli* ATCC8739, where the observed antibacterial value R and reduction in percentages of *E. coli* was lower compared to our findings for Ti CB (>2.8 and 99.4% vs >3.7 and 100%), despite using the same international standards and experimental methodology. However, their inoculation amount of CFUs seemed to be tenfold higher than ours, possibly explaining the lower reduction values. Apparent reductions <99.9% arose because the uncoated controls yielded too few CFU after 24 hours, with no detectable CFUs on SML-coated discs, the calculation is capped by the assay’s detection limit and the small control count, indicating a detection-limited denominator rather than residual survival. The percentages of reduction of *S. aureus* and *S. epidermidis* were similar, however the antibacterial R values of *S. aureus* and *S. epidermidis* were higher in their study compared to our findings for Ti CB (>4.0 and >3.7 vs >3.1 and >2.4 respectively). The latter could be explained due to the difference between surface roughness. Additional evidence from *in vivo* models supports the antibacterial performance of the SML coating, as Fabritius et al., 2020, demonstrated protection against *S. epidermidis* in a rabbit tibial implant model (Fabritius et al., 2020). Similarly, coatings incorporating silver have achieved complete eradication of bacterial growth (Kalishwaralal et al., 2010; Medina et al., 2025). Taken together, these data demonstrate that the SML coating provides robust antibacterial protection across diverse bacterial species on both CoCr and Ti substrates with various roughness.

In contrast to the lack of bacterial growth on the coated discs, uncoated control discs supported bacterial growth and surface-dependent differences were observed. *S. epidermidis* bacterial growth was more abundant on the Ti CB surface compared to the Ti polished surface. This is in line with a previous reported literature by Kuik et al., 2025, exhibiting that CB discs facilitated more bacterial growth than polished discs (Kuik et al., 2025). Nano- or micro-patterned structures of surfaces like spikes can influence the adhesion of bacteria, and have been shown to possess bactericidal effects on *E. coli*, *S. aureus* and *S. epidermidis* (Hsu et al., 2013; May et al., 2015; Francone et al., 2021). However, CB discs used in this study did not contain nano- or micro-patterned structures, and it has been shown that in general ultra-smooth surfaces (Ra <0.2) might be unfavorable for bacteria to adhere to, or to accumulate biofilms on (Teughels et al., 2006; Ribeiro et al., 2012). In addition to that, surface wettability also plays a role in bacterial adhesion to implant materials (Yang et al., 2022). In general, more hydrophilic surfaces with lower contact angles favor bacterial attachment by facilitating interactions between microbial cells and the substrate, though this is dependent on bacterial species and materials (Gallardo-Moreno et al., 2009). Nanostructured titanium surfaces with increased hydrophilicity have been shown to promote adhesion of *E. coli* under *in vitro* conditions (Lorenzetti et al., 2015). Previous unpublished wettability tests showed that the Ti CB discs were considered hydrophilic, while the Ti polished were considered hydrophobic. This possibly explains the higher abundance of bacterial growth on the CB discs as well.

Even though the uncoated polished samples did not visually show bacterial adhesion using SEM imaging, the uncoated CB samples did show visual bacterial adhesion using SEM imaging. No consistent reduction in CFU counts was observed on the polished discs compared to the CB discs, indicating that the SEM observations and CFU results were not entirely in agreement. This discrepancy may be attributed to differences in surface topography, as previously discussed that smoother polished surfaces have been shown to provide less anchoring sites for bacterial attachment than the rougher CB surfaces (Ribeiro et al., 2012; Zheng et al., 2021). Additionally, the apparent absence of attached bacteria on the polished discs in SEM images could be explained by the detachment of loosely adhered bacterial cells or biofilm fragments during the sample preparation steps required for SEM imaging. SEM images indicate the presence of debris or possible remnants of bacterial cells in SML-coated samples of both polished as well as CB samples, therefore confirming the antibacterial activity of the SML coating present on at least the CB discs. Similar findings have been shown previously, in a study of Azab et al., 2016, where there were also no bacterial cells visible anymore on SEM images after 24 hours of incubation on a Ti disc with a silver-impregnated coating (Azab et al., 2016). Seemingly, some extracellular matrix that was left could be seen, possibly indicating a remainder of initial biofilm formation. It has been previously described that after one hour of incubation, the SML coating is not yet able to become effectively antibacterial (van Hoogstraten et al., 2024).

For both *S. aureus* and *E. coli*, it was seen that bacteria grew better on Ti than CoCr. Similarly, *S. epidermidis* showed the most bacterial growth on Ti CB. Watanabe et al., 2021, also used the JIS 2801 standard and found similar results where *S. aureus* NBRC 12732 and *P. acnes* NBRC 107605 grew less well on CoCr alloys compared to Ti alloys both *in vitro* and *in vivo* (Watanabe et al., 2021). Koseki et al., 2014, grew *S. epidermidis* ATCC35984 *in vitro* for six hours, and found that they grew better on Ti compared to CoCr, analyzed by crystal violet staining and CFU counts (Koseki et al., 2014). Similarly, McGaffey et al., 2019 used 3D printed metals and reported more biofilm growth on Ti alloy compared to CoCr alloy (McGaffey et al., 2019). In general, our data suggests that CoCr would be favorable as material since it facilitates less bacterial growth after 24 hours of incubation. Conversely, clinical data remain less clear, where Wright et al., 2016 (Wright et al., 2016) reported no statistical significant differences in infection risk between implant materials in spinal instrumentation, while Patel et al., 2016 (Patel et al., 2016) described enhanced biofilm formation on CoCr compared to Ti under non-standardized conditions. Methodological differences such as incubation times, inoculum concentration, and absence of standardized assays complicate direct comparisons across studies.

This study has several limitations. Firstly, all experiments were performed under static *in vitro* conditions, which do not replicate the complex physiological environment of the joint, including immune responses and fluid dynamics. However, the static model was chosen as test model in this study, as the international standards which were used in this study specify static conditions. Additionally, a previous study has shown that after 7-days of *Pseudomonas putida* incubation on discs with the SML-coating in a dynamic CDC bioreactor model, mainly dead bacteria were found in the biofilm (Khalilpour, 2009). Secondly, mainly ATCC reference strains were tested. It is not expected that clinical isolates would respond similarly to ATCC reference strains, due to their often heterogeneous and resilient character (Trobos et al., 2022). For instance, *P. aeruginosa* ATCC15442 used in this study is a non-pathogenic environmental bacterial strain (Wang et al., 2014), previously used in disinfectant testing (Romao et al., 2005). It was also used in the ASTM E-2180–24 test standard which was used in this study as well, therefore this strain was selected (ASTM E 2180-24, 2024). *P. aeruginosa* ATCC15442 is known to produce strong biofilms which was also seen from the attachment assays where the biomass amount was similar to that of another strong biofilm former (*P. aeruginosa* ATCC15692) (Berkl et al., 2022), however it has a limited clinical link to PJI (Paldrychova et al., 2020). Therefore, a clinical *P. aeruginosa* strain isolated from a PJI was included in the testing as well. Including this clinical strain did show that the SML coating is also potent against clinical PJI related strains, and not only ATCC strains. Finally, this study does not allow for conclusions to be drawn regarding long-term antibacterial activity due to conducting measurements only after 24 hours, essentially only assessing bacterial adhesion and initial biofilm formation.

Despite these limitations, these first results strongly support the potential of the SML coating to prevent early bacterial colonization and subsequent biofilm formation on implant materials. Future work should extend these observations to clinical PJI isolates, *in vivo* infection models, and ultimately clinical studies to confirm efficacy under physiological conditions. The SML coating could represent a valuable addition to prevention strategies of bacterial colonization in prosthetic joint surgery by combining the strong antibacterial activity with previously demonstrated biocompatibility and osseointegration.

## 6 Conclusion

New strategies to prevent bacterial colonization of orthopedic implants are needed to enhance patient safety in joint replacement surgery. This study aimed to evaluate the efficacy of a novel silver multilayer coating. The silver coating clearly demonstrated persuasive and potent antibacterial activity against both Gram-positive and Gram-negative species, with reductions exceeding 99.2% in bacterial counts. Antibacterial activity (R value), similar to CFU log_10_ reduction, was >2.6 ± 0.3 for *S. aureus* ATCC6538p, >1.9 ± 0.2 for *S. epidermidis* ATCC35984, >2.6 ± 0.6 for *E. coli* ATCC8739, >3.2 ± 0.4 for *P. aeruginosa* ATCC15442, and >2.1 ± 0.8 for the clinical PJI *P. aeruginosa* strain. Notably, *S. aureus* ATCC6538p and *E. coli* ATCC8739 exhibited higher levels of growth on Ti discs compared to CoCr discs. For *P. aeruginosa* ATCC15442, the lowest level of bacterial growth was observed on the CoCr CB surface, while *S. epidermidis* grew more extensively on the Ti CB surface, compared to the other surfaces. This indicates that CoCr facilitates less bacterial growth, and might be favorable as implant material, although other factors such as ductility, fatigue strength, and osseointegration must also be considered in material selection for orthopedic applications. The SML coating is considered a promising strategy to prevent bacterial adhesion and colonization on orthopedic implants in regards to PJI potentially limiting biofilm formation and maturation. Future studies will include both *in vitro* and *in vivo* experiments using clinical isolates to further assess the antimicrobial performance of the SML coating.

## Data Availability

The raw data supporting the conclusions of this article will be made available by the authors, without undue reservation.
