# Supplementary material for: Silver multilayer coating on orthopedic implant material of different alloys and surfaces significantly reduces bacterial colonization
Source: Front Cell Infect Microbiol. 2025 Nov 26;15:1707694. doi: 10.3389/fcimb.2025.1707694 (PMC12689873; doi:10.3389/fcimb.2025.1707694)
Supplement: Supplementary file 1 [file Table1.docx]

Supplementary data to:

Silver multilayer coating on orthopedic implant material of different alloys and surfaces significantly reduces bacterial colonization

L.T.D. Speijker, J. Fechter, R. Bargon, J. Dingemans, J.J. Arts, P.H.M. Savelkoul, I.H.M. van Loo

# Results

## Effects of SML coating and surface type on clinical PJI *P. aeruginosa* bacterial growth

All SML-coated discs (Ti and CoCr) showed <50 CFUs per sample for the clinical PJI *P. aeruginosa* strain, indicating a reduction in bacterial load of more than >99.2% compared to uncoated controls (*p*<0.05; Supplementary figure 1). Significantly higher CFU counts were found on Ti compared to CoCr regarding the polished surface (n=3 per surface). More specifically, the clinical PJI *P. aeruginosa* strain showed an antibacterial activity (R) and CFU log_10_ reduction greater than 2.1±0.8 (99.2%) across all surfaces (Supplementary Table 1).


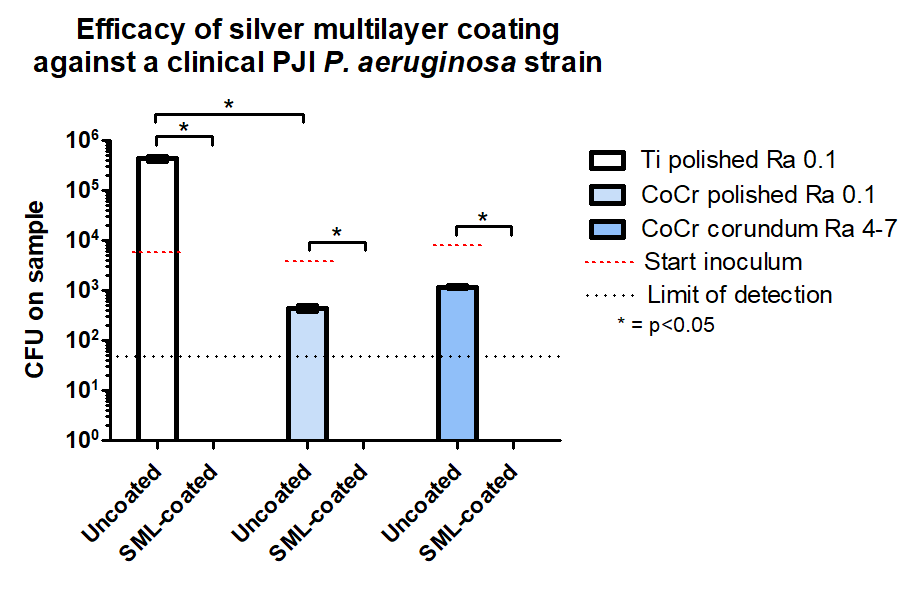


Supplementary figure 1: Efficacy of SML coating, surface, and material on the reduction of bacterial growth of the clinical PJI P. aeruginosa strain. Bars represent the mean colony-forming units (CFU) of an experiment performed in triplicate (n=3) per surface, except for CoCr CB in duplicate (n=2). Error bars indicate the standard error of the mean. Statistical analysis was performed using the Kruskal-Wallis test, with post hoc analysis (Mann-Whitney U test). * = p<0.05. Abbreviations: SML = silver multilayer; Ti = titanium alloy; CoCr = cobalt-chromium-molybdenum alloys; CB = corundum blasted; Ra = surface roughness average; ATCC = American Type Culture Collection; PJI = prosthetic joint infection

Supplementary table 1: Overview of the bacterial reduction (%) and antibacterial activity (R) for the clinical PJI P. aeruginosa strain.

| Micro-organism | Disc type | Reduction in viable bacteria  (uncoated vs. SML-coated) in % | R (antibacterial activity) |
| --- | --- | --- | --- |
| *P. aeruginosa* clinical PJI strain | Ti polished Ra 0.1 μm | 100.0 | >3.9 |
|  | CoCr polished Ra 0.1 μm | >99.2 | >0.9 |
|  | CoCr CB Ra 4-7 μm | >99.7 | >1.4 |

*Values represent the reduction in viable bacteria of* one *experiment* performed in triplicate*, comparing uncoated*  *and SML coated discs* (n=3, except for CoCr CB n=2)*. Abbreviations:* PJI = prosthetic joint infection; *SML = silver multilayer; Ti = titanium alloy; CoCr = cobalt-chromium-molybdenum alloys;* CB = corundum blasted; *Ra = surface roughness average; ATCC = American Type Culture Collection.*

## Scanning electron microscopy (SEM)

Supplementary figure 2 shows the incubation of *S. aureus, S. epidermidis, P. aeruginosa* and *E. coli* on uncoated discs. Polished surfaces exhibited visible bacterial growth only for *S. aureus* ATCC6538p, whereas no growth was observed for the other tested species on either Ti or CoCr substrates. In contrast, Supplementary figure 3 demonstrates that the CB surfaces did visibly facilitate bacterial growth for all tested bacterial species, independent of the underlying material.

A) *S. aureus* ATCC6538p


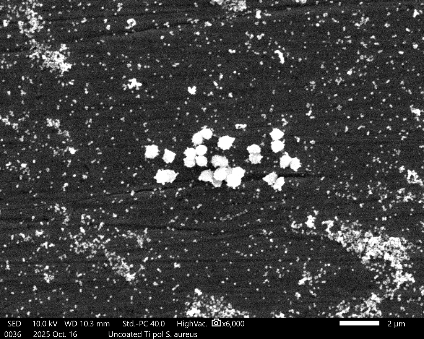


B) *S. epidermidis* ATCC35984


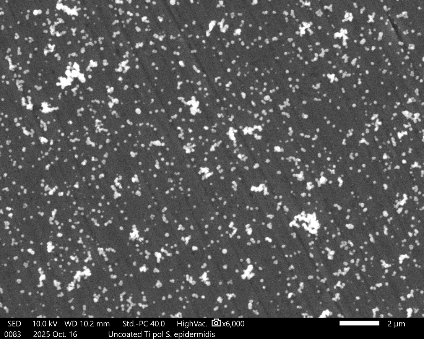

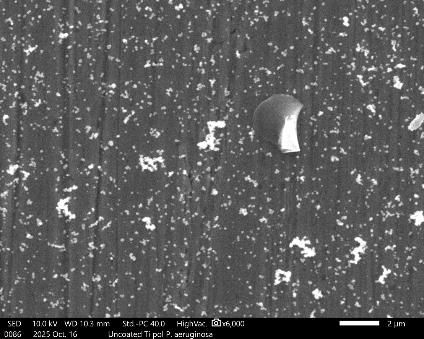


C) *P. aeruginosa* ATCC15442


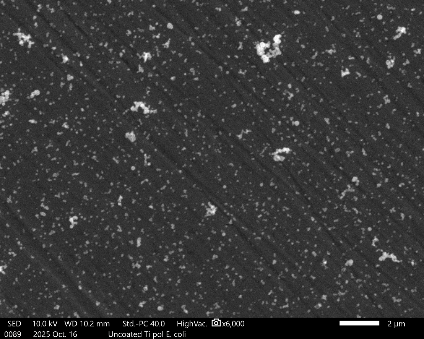


D) *E. coli* ATCC8739


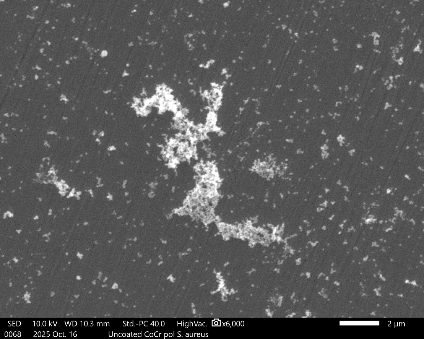

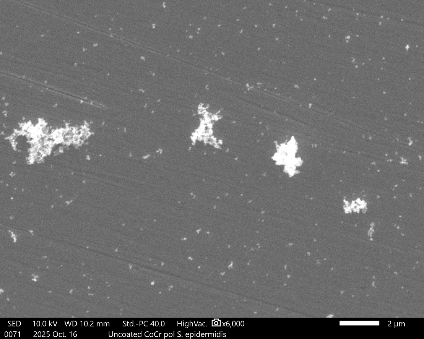

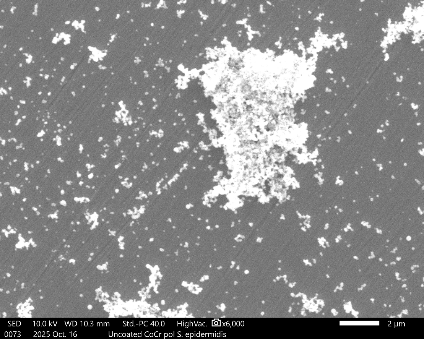

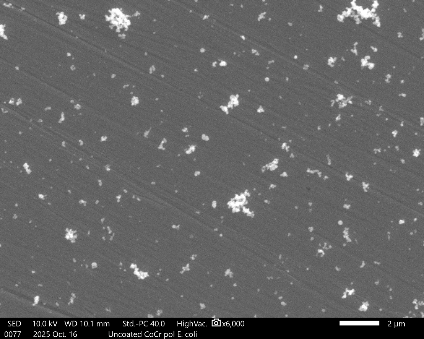


Ti polished Ra 0.1

CoCr polished Ra 0.1

Supplementary figure 2: Scanning electron microscopy (SEM) visualization of S. aureus ATCC6538p, S. epidermidis ATCC35984, P. aeruginosa ATCC15442, and E. coli ATCC8739 survival on different uncoated materials (Ti polished Ra 0.1, and CoCr polished Ra 0.1, n=1 per condition) to assess the ability to image cells. Orange arrows indicate bacterial cells, while white arrows indicate debris or remnants of bacterial cells or biofilm matrix. Images were acquired at 6000X magnification, with scale bars representing 2 µm. Abbreviations: SML = silver multilayer; Ti = titanium alloy; CoCr = cobalt-chromium-molybdenum alloys; Ra = surface roughness average;


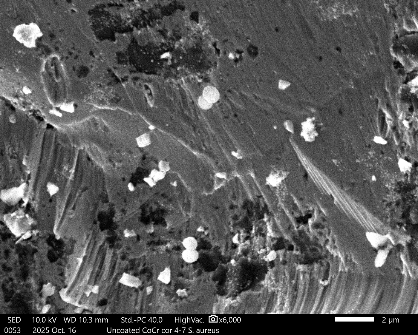

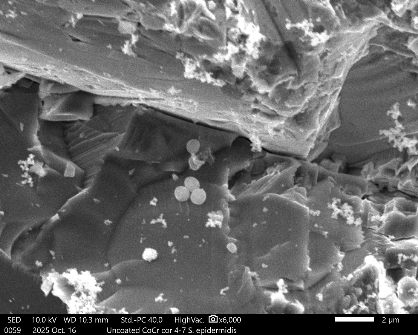

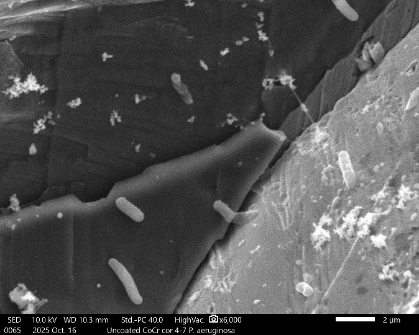

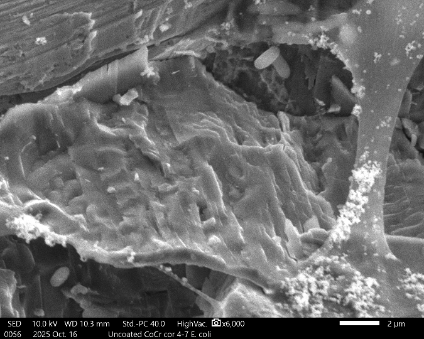


CoCr corundum blasted Ra 4-7


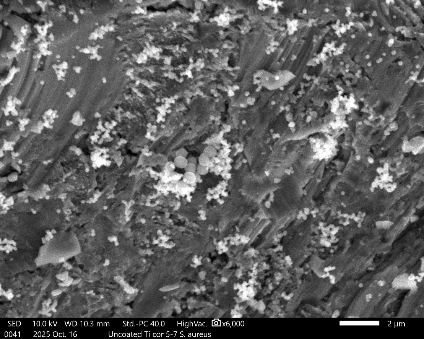

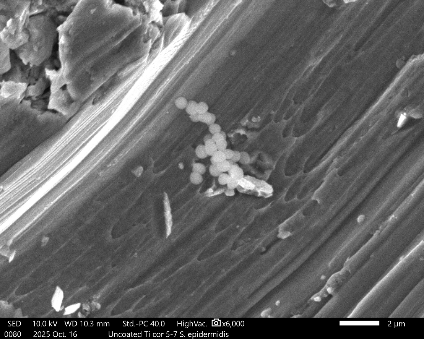

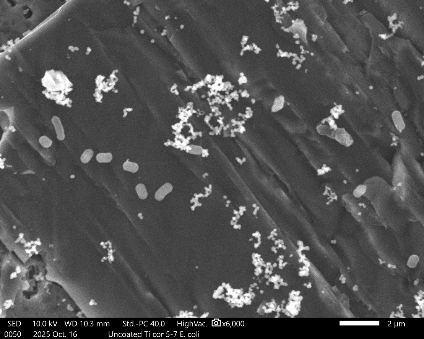

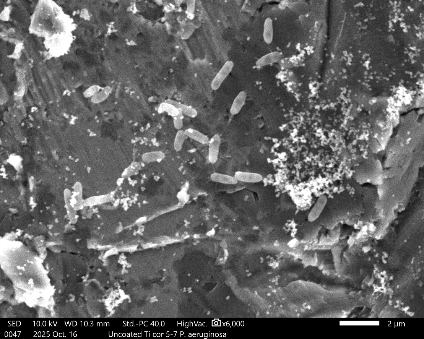


Ti corundum blasted Ra 5-7

A) *S. aureus* ATCC6538p

B) *S. epidermidis* ATCC35984

C) *P. aeruginosa* ATCC15442

D) *E. coli* ATCC8739

Supplementary figure 3: Scanning electron microscopy (SEM) visualization of S. aureus ATCC6538p, S. epidermidis ATCC35984, P. aeruginosa ATCC15442, and E. coli ATCC8739 survival on different uncoated materials (ti polished Ra 0.1, and CoCr polished Ra 0.1, n=1 per condition) to assess the ability to image bacterial cells. Orange arrows indicate bacterial cells, while white arrows indicate debris or remnants of bacterial cells or biofilm matrix. Images were acquired at 6000X magnification, with scale bars representing 2 µm. Abbreviations: SML = silver multilayer; Ti = titanium alloy; CoCr = cobalt-chromium-molybdenum alloys; Ra = surface roughness average; Ra = surface roughness average
